# Identification of BST2 as a conjunctival epithelial stem/progenitor cell marker

**DOI:** 10.1016/j.isci.2023.107016

**Published:** 2023-06-05

**Authors:** Masahiro Kitao, Ryuhei Hayashi, Kimihito Nomi, Reiko Kobayashi, Tomohiko Katayama, Hiroshi Takayanagi, Akiko Oguchi, Yasuhiro Murakawa, Kohji Nishida

**Affiliations:** 1 Department of Ophthalmology, Osaka University Graduate School of Medicine, Suita, Osaka 565-0871, Japan; 2 Department of Stem Cells and Applied Medicine, Osaka University Graduate School of Medicine, Suita, Osaka 565-0871, Japan; 3 Integrated Frontier Research for Medical Science Division, Institute for Open and Transdisciplinary Research Initiatives, Osaka University, Suita, Osaka 565-0871, Japan; 4 Business Strategy Office, ROHTO Pharmaceutical, Osaka, Osaka 544-0012, Japan; 5 RIKEN-IFOM Joint Laboratory for Cancer Genomics, RIKEN Center for Integrative Medical Sciences, Yokohama, Kanagawa 230-0045, Japan; 6 Institute for the Advanced Study of Human Biology (ASHBi), Kyoto University, Kyoto, Kyoto 606-8501, Japan; 7 Premium Research Institute for Human Metaverse Medicine (WPI-PRIMe), Osaka University, Suita, Osaka 565-0871, Japan

**Keywords:** Cell biology, Stem cells research, Transcriptomics

## Abstract

The conjunctival epithelium consists of conjunctival epithelial cells and goblet cells derived from conjunctival epithelial stem/progenitor cells. However, the source of these cells is not well known because no specific markers for conjunctival epithelial stem/progenitor cells have been discovered. Therefore, to identify conjunctival epithelial stem/progenitor cell markers, we performed single-cell RNA sequencing of a conjunctival epithelial cell population derived from human-induced pluripotent stem cells (hiPSCs). The following conjunctival epithelial markers were identified: BST2, SLC2A3, AGR2, TMEM54, OLR1, and TRIM29. Notably, BST2 was strongly positive in the basal conjunctival epithelium, which is thought to be rich in stem/progenitor cells. Moreover, BST2 was able to sort conjunctival epithelial stem/progenitor cells from hiPSC-derived ocular surface epithelial cell populations. BST2-positive cells were highly proliferative and capable of successfully generating conjunctival epithelial sheets containing goblet cells. In conclusion, BST2 has been identified as a specific marker of conjunctival epithelial stem/progenitor cells.

## Highlights

• Single-cell RNA-seq of hiPSC-derived conjunctival epithelial cells revealed BST2
• BST2 strongly expressed in the basal conjunctival epithelium rich in stem cells
• BST2 can be used to sort conjunctival epithelial stem cells
• BST2-positive cells generated conjunctival epithelial sheet containing goblet cells

## Graphical abstract

**Figure:**
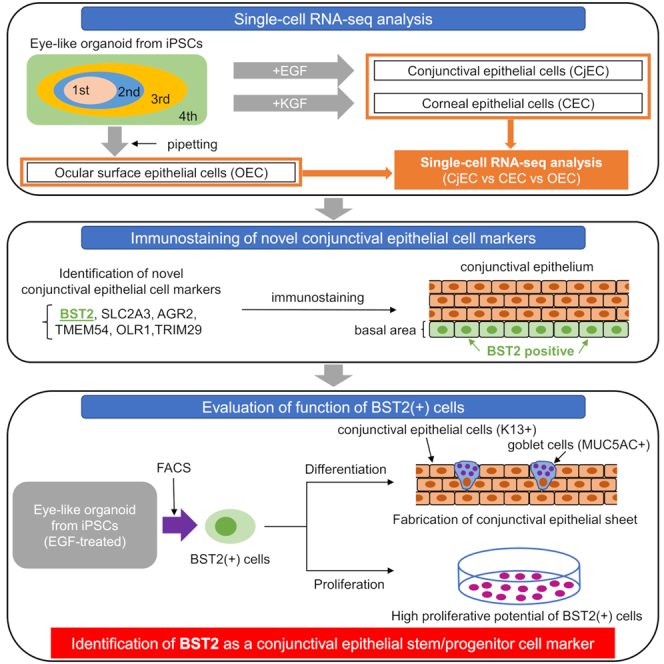

## Introduction

The ocular surface comprised the conjunctival epithelium, corneal epithelium, lacrimal glands, meibomian glands, and tear film, all of which play essential roles in maintaining good vision.^1^ The conjunctiva, which covers the sclera and the posterior surface of the eyelids, facilitates smooth eye movement and protects the ocular surface by regulating the immune response.^2^ The conjunctival epithelium also contains goblet cells that produce mucin, an essential component of the tear film.^3^ Despite substantial advances in corneal epithelium research, much remains unknown about the conjunctival epithelium. There is abundant evidence, for example, that corneal epithelial stem/progenitor cells exist at the limbus, with several limbal epithelial stem/progenitor cell markers previously reported.^4^ From a clinical perspective, limbal stem cell insufficiency can be treated using a variety of regenerative therapies, including cultivated corneal epithelial sheets, oral mucosal epithelial sheets, and human-induced pluripotent stem cell (hiPSC)-derived corneal epithelial sheets.^5^^,^^6^ However, the source of conjunctival epithelial stem/progenitor cells remains controversial and no specific markers for conjunctival epithelial stem/progenitor cells have yet been identified.^7^ Moreover, although conjunctival epithelial stem/progenitor cells are believed to be abundant in the conjunctival fornix, a full appreciation of their localization and the molecular mechanisms that affect their behavior remain unclear.^8^ It is also more difficult to obtain tissue from the conjunctival epithelium compared to the corneo-scleral limbal epithelium, which hinders research on the conjunctival epithelium, and relatively few regenerative therapies exist for the conjunctival epithelium because conjunctival epithelial sheets cannot currently be fabricated with high purity.^7^

Recently, we generated hiPSC-derived self-formed ectodermal autonomous multi-zone (SEAM) organoids that mimic whole-eye development.^9^^,^^10^ In each of four concentric cellular zones, cells had characteristics of cells that comprise different ocular component tissues such as the lens, neuro-retina, ocular surface epithelium, and lacrimal gland.^11^ We also succeeded in isolating and differentiating conjunctival epithelial stem/progenitor cells and corneal epithelial stem/progenitor cells, respectively, via different differentiation culture methods. Conjunctival epithelial stem/progenitor cells, for example, preferentially emerged in epidermal growth factor (EGF)-treated SEAMs, whereas corneal epithelial stem/progenitor cells predominated in keratinocyte growth factor (KGF)-treated SEAMs.^12^ There are no known conjunctival epithelial stem/progenitor cell markers, especially cell surface markers, capable of separating conjunctival epithelial stem/progenitor cells from corneal epithelial stem/progenitor cells, and hiPSC-derived conjunctival epithelial sheets made using conventional SEAM cultivation methods occasionally contain some corneal epithelial cells, with a lack of goblet cells. Identifying a new conjunctival epithelial stem/progenitor cell marker would allow cell sorting and enable the generation of high-purity conjunctival epithelial sheets to contribute to the development of regenerative medicine applications and drug discovery as applied to the conjunctival epithelium.

Currently, single-cell RNA sequencing (scRNA-seq) has completely changed our understanding of diseases and associated biological processes. This technology has led to the discovery of new cell types, unique cell states, and novel tissue-specific genes, including stem/progenitor cell markers.^13^ In a recent study, scRNA-seq was used to identify limbal/corneal epithelial stem/progenitor cell markers in the human limbus.^14^ However, few studies have employed scRNA-seq to research conjunctival epithelial stem/progenitor cell markers owing to the difficulty of obtaining human conjunctiva, especially the conjunctival fornix, which are predicted to be rich in conjunctival epithelial stem/progenitor cells. To overcome this problem, we performed scRNA-seq on hiPSC/SEAM-derived conjunctival epithelial stem/progenitor cell populations to identify new conjunctival epithelial stem/progenitor cell markers, especially cell surface markers, that could be used to isolate conjunctival epithelial stem/progenitor cells from corneal epithelial stem/progenitor cells, similarly analyzed by scRNA-seq. A comparison of the conjunctival and corneal epithelial stem/progenitor scRNA-seq data obtained from hiPSC/SEAM-derived cell populations identifies several novel conjunctival epithelial cell markers, including one stem/progenitor cell marker, BST2.

## Results

### Single-cell RNA sequencing of ocular surface epithelial cells, conjunctival epithelial cells, and corneal epithelial cells derived from hiPSCs

**Figure 1:**
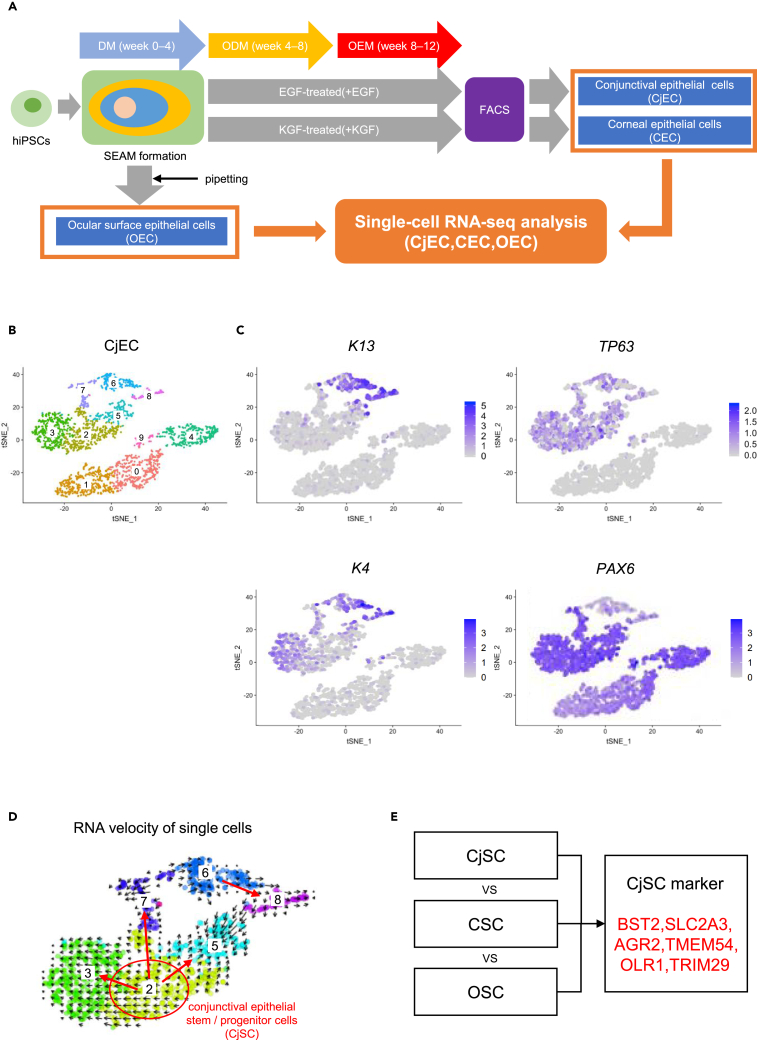
Single-cell RNA sequencing of conjunctival epithelial cells derived from hiPSCs via SEAMs (A) Schematic of differentiation methods for ocular surface epithelial cells, conjunctival epithelial cells, and corneal epithelial cells: DM; differentiation medium, ODM; ocular surface differentiation medium, OEM; ocular surface epithelium maintenance medium. (B) t-distributed stochastic neighbor embedding of conjunctival epithelial cells analyzed by single-cell RNA sequencing. 10 cell clusters were generated. (C) Feature plots of K13, K4, TP63, and PAX6. (D) RNA velocity of single cells of conjunctival epithelial cells. Clusters 2, 3, 5, 6, 7, and 8 were extracted. Each arrow shows the direction of differentiation. (E) Schematic of the detection of conjunctival epithelial stem/progenitor cell markers. See also Figure S1.

hiPSCs were cultivated for four weeks, during which time they spontaneously formed typical SEAMs with four identifiable concentric zones. Each SEAM exhibited four concentric cellular zones: cells in the innermost zone-1 were similar to the neuroectoderm; cells in zone-2, more peripherally, most closely resembled those of the optic cup and neural crest; cells in zone-3, more peripherally again, contained cells of the ocular surface ectoderm and lens; and cells in the outermost zone-4 had characteristics of the surface ectoderm, including skin. Cells in SEAM zones-3 and -4 at four weeks’ cultivation were identified as a group of ocular surface epithelial cells (OEC) containing stem/progenitor cells. At this juncture, zone-1 and -2 cells were removed from the SEAMs by manual pipetting and EGF or KGF was added to the culture medium to induce the emergence of OEC of a conjunctival or corneal epithelial cell lineage, respectively (Figures 1A and S1A). After 12 weeks of cultivation, EGF-treated SEAMs (minus zones 1 and 2) were subjected to fluorescent-activated cell sorting (FACS) as described previously,^12^ with the CD200-/CD104+/SSEA4weakly+ cell population containing stem/progenitor cells designated as conjunctival epithelial cells (CjEC) (Figures 1A and S1B). KGF-treated SEAMs at 12 weeks were also subjected to FACS as described previously,^9^^,^^15^ with the CD200-/CD104+/SSEA4+ cell population containing stem/progenitor cells designated as corneal epithelial cells (CEC) (Figures 1A and S1C).

scRNA-seq analysis was performed and t-distributed stochastic neighbor embedding data for CjEC, CEC, and OEC were obtained (Figures 1B, S1D, and S1G). We first considered CjEC, and Figure 1C shows feature plots of K13 (a mature conjunctival epithelial cell marker), K4 (a mature conjunctival epithelial cell marker), TP63 (an epithelial stem cell marker), and PAX6 (an ocular surface epithelial cell marker). Clusters of CjEC were then classified, with clusters 2, 3, 5, and 7 identified as immature conjunctival epithelial cells and clusters 6 and 8 as mature conjunctival epithelial cells. RNA velocity, a high-dimensional vector that can predict the future state of individual cells on a temporal scale,^16^ was also used to investigate the developmental lineages of the immature conjunctival epithelial cell population. This determined that CjEC cluster 2 comprised the conjunctival epithelial stem/progenitor cell population (Figure 1D). A further investigation of the highly expressed markers in CjEC cluster 2 that were simultaneously absent or expressed at low levels in other clusters identified BST2, SLC2A3, AGR2, TMEM54, OLR1, and TRIM29 (Figure 1E). Accordingly, these are proposed as novel conjunctival epithelial cell markers, all of which are cell surface markers, which could be useful for FACS analysis and cell sorting.

We similarly analyzed the feature plots of K12 (a mature corneal epithelial cell marker), K3 (a mature corneal epithelial cell marker), TP63, and PAX6 expression in CEC (Figure S1E), and classified clusters 0, 1, 2, 9, and 10 as immature corneal epithelial cells and clusters 3, 4, 5, 6, 7, and 8 as mature corneal epithelial cells. An analysis of the RNA velocity further determined that CEC cluster 0 comprised the corneal epithelial stem/progenitor cell population (Figure S1F). An analysis of the feature plots of TP63 and PAX6 expression in OEC determined that cluster 1 comprised the ocular surface epithelial stem/progenitor cell population (Figure S1H).

### Immunolocalization of novel conjunctival epithelial cell markers and stem/progenitor cell marker

**Figure 2:**
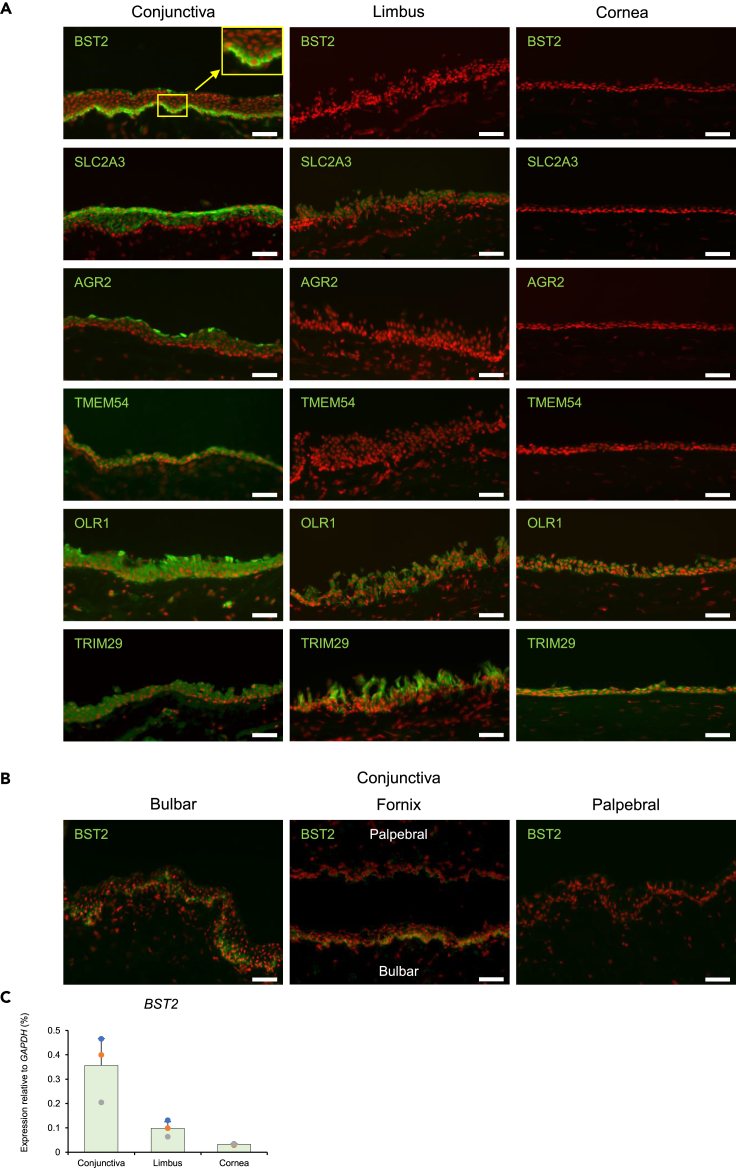
Novel conjunctival epithelial cell markers (A) Immunostaining of human conjunctiva, limbus, and cornea with antibodies to BST2, SLC2A3, AGR2, TMEM54, OLR1, and TRIM29 (green). (B) Immunostaining of cynomolgus monkey bulbar, fornix, and palpebral conjunctiva with an antibody to BST2 (green). (C) BST2 gene expression of human conjunctival, limbal, and corneal epithelium. n = 3. Error bars show the standard deviation. (A and B) Nuclei, red. Scale bars; 50 μm. See also Figure S2.

Tissue sections of human conjunctiva, limbus, and cornea were immunostained with antibodies to each of the proposed conjunctival epithelial cell markers, BST2, SLC2A3, AGR2, TMEM54, OLR1, and TRIM29, and all were found to strongly stain the human bulbar conjunctival epithelium (Figure 2A). Of these, BST2, AGR2, and TMEM54 were not identified in the limbal or corneal epithelium. SLC2A3 was identified in the conjunctival and limbal epithelium but not in the corneal epithelium, whereas OLR1 and TRIM29 stained the conjunctival, limbal, and corneal epithelium. Of the novel conjunctival epithelial markers, BST2 was notable for its positive labeling of the basal conjunctival epithelium. As we could not obtain human fornix conjunctiva for this study, we analyzed tissue from a cynomolgus monkey and found that basal regions of both the bulbar and fornix conjunctival epithelia were positive for BST2, especially the basal region of the fornix conjunctiva on the bulbar side (Figure 2B). All other markers also labeled monkey fornix conjunctiva (Figure S2). A gene expression analysis confirmed that *BST2* was upregulated in the human bulbar conjunctival epithelium, compared to the limbal or corneal epithelia (Figure 2C). Of the novel conjunctival epithelial markers, BST2, AGR2, and TMEM54 are conjunctival epithelium-specific. Among them, only BST2 exclusively immunostained the basal layer of the conjunctival epithelium, suggesting it to be the most representative marker of conjunctival epithelial stem/progenitor cells^7^ (Figure 2A). AGR2 did not stain the basal layer, while TMEM54 stained not only the basal layer but also cells in the outer conjunctival layers, so both differed from the typical immunostaining patterns of conjunctival epithelial stem/progenitor cells. Accordingly, we excluded AGR2 and TMEM54 from our ongoing studies, and shifted our focus to BST2.

### Proliferative potential of FACS-sorted EGF-treated SEAMs

**Figure 3:**
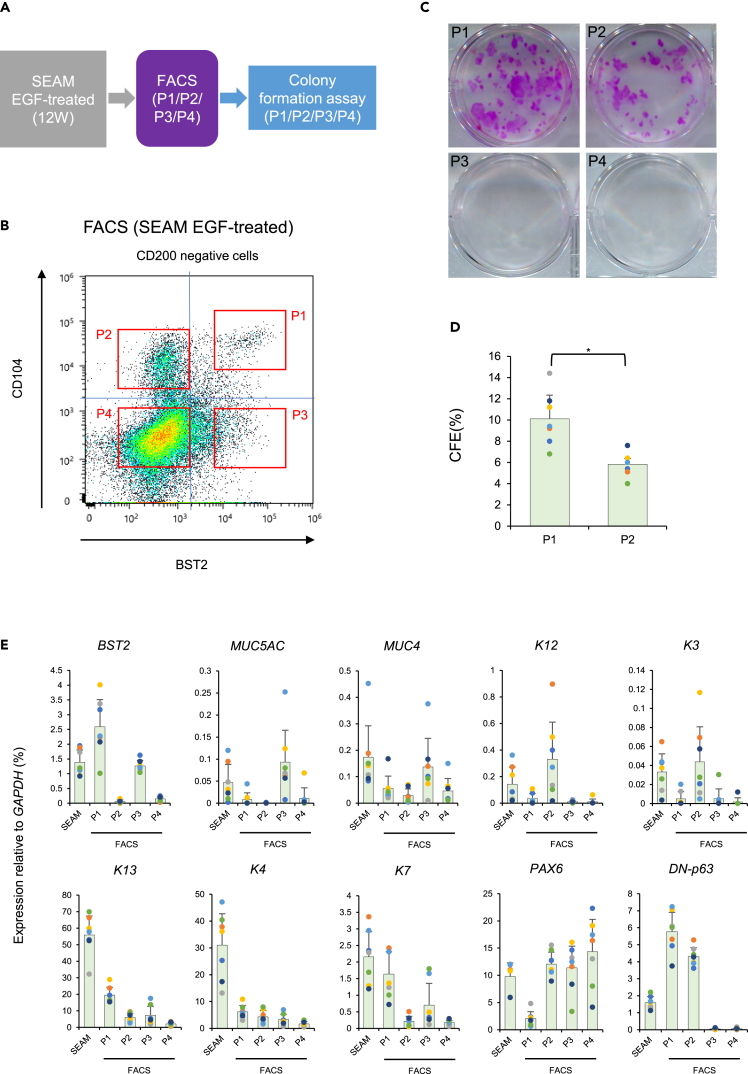
Proliferative potential of hiPSC/SEAM-derived BST2+ cells (A) Schematic of the differentiation and sorting method for the cells. (B) Flow cytometric analysis of CD200, CD104, and BST2 for the EGF-treated SEAM derivative at week 12. CD200- cells were extracted first, and then the P1–P4 fractions were sorted based on CD104 and BST2 expression. P1–P4 fractions are defined as follows: (P1) CD200-, CD104+, and BST2+ cells; (P2) CD200-, CD104+, and BST2-cells; (P3) CD200-, CD104-, and BST2+ cells; (P4) CD200-, CD104-, and BST2- cells. (C) Representative images of colony-forming assays of sorted cells from P1, P2, P3, and P4. n = seven independent experiments. (D) Colony-forming efficiency of sorted cells from P1 and P2. n = 7. ∗p < 0.05. Error bars show the standard deviation. (E) Gene expression analysis of ocular surface epithelial cell markers in the EGF-treated SEAM derivative at week 12 and in P1, P2, P3, and P4 cells. n = 7. ∗p < 0.05. Error bars show the standard deviation. See also Figures S3 and S4.

As mentioned, EGF-treated SEAMs contain conjunctival epithelial stem/progenitor cells. We immunostained EGF-treated SEAMs at week 6 and week 12 of development to verify the expression and location of BST2. Immunostaining of EGF-treated SEAMs at week 6 showed that BST2-positive cells mainly existed in zones 3 and 4, which contain ocular surface epithelial stem/progenitor cells^9^^,^^15^ (Figure S3A). Next, we immunostained EGF-treated SEAMs at week 12 and observed BST2-positive cells, which we assume contain conjunctival epithelial stem/progenitor cells (Figure S3B). To assess the proliferative potential of BST2+ CjEC, we subjected EGF-treated SEAM cells at week 12 to FACS to isolate conjunctival epithelial stem/progenitor cells using CD200, CD104, and BST2. P1–P4 cells were compared via colony-forming assays (CFA) and gene expression analysis (Figures 3A and 3B), with P1–P4 defined as follows: P1, CD200-/CD104+/BST2+ cells; P2, CD200-/CD104+/BST2- cells; P3, CD200-/CD104-/BST2+ cells; P4, CD200-/CD104-/BST2- cells.

CFA disclosed that P1 and P2 cells had a high proliferative potential, whereas P3 and P4 cells had no proliferative potential. Cells from P1, moreover, were larger and formed more colonies than cells from P2 (Figure 3C), indicating that the colony-forming efficiency (CFE) of P1 was significantly higher than that of P2 (Figure 3D). These findings indicated that P1 or P2 cells (i.e. CD104+ cells) contain conjunctival epithelial stem/progenitor cells, whereas P3 and P4 cells (i.e. CD104-) do not. Overall, P1 cells (i.e. those that are CD104+/BST2+) had the highest proliferative potential among P1–P4 cells. Although this result was obtained using the YZWJs524 hiPSC line, we have also confirmed, using line 201B7, that P1 cells had the highest proliferative potential among P1–P4 cells (Figures S4A–S4C).

Gene expression analysis revealed that cells in the CD104+ fraction (P1 and P2) exhibited elevated expression of *DN-p63* (epithelial stem cell markers). This confirmed the CFA results that P1 and P2 had high proliferative potential. Gene expression analysis also revealed that *K13* and *K4* (conjunctival epithelial cell markers) and *K7* (unicellular gland marker) were elevated in P1, whereas *K12* and *K3* (corneal epithelial cell markers) were elevated in P2. We speculated that P1 contained conjunctival epithelial stem/progenitor cells, and that P3 contained differentiated conjunctival epithelial cells because *MUC5AC* (a goblet cell marker) and *MUC4* (a membrane mucin marker) were elevated in P3 (Figure 3E).

### Fabrication of epithelial sheets from hiPSC-derived BST2+ cells

**Figure 4:**
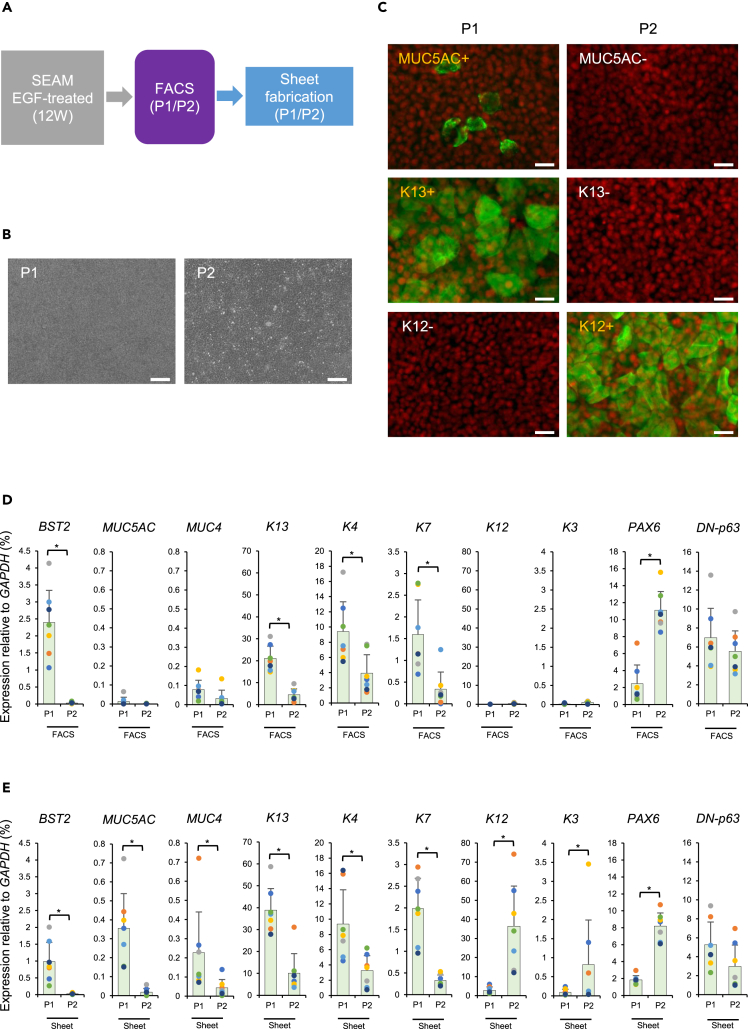
Fabrication of epithelial sheets from hiPSC/SEAM-derived BST2+ cells (A) Schematic of the method of generating epithelial sheets from the cells. (B) Phase-contrast images of the sheet fabricated from sorted BST2+ (P1) and BST2- (P2) cells. n = three independent experiments. Scale bars; 100 μm. (C) Immunostaining of MUC5AC, K13, and K12 (green) in the sheet derived from P1–P2 cells. n = three independent experiments. Nuclei, red. Scale bars; 25 μm. (D) Gene expression analysis of ocular surface epithelial cell markers for sorted cells from P1–P2 (FACS). n = 7. ∗p < 0.05. Error bars show the standard deviation. (E) Gene expression analysis of ocular surface epithelial cell markers for the sheet derived from P1–P2 (Sheet). n = 7. ∗p < 0.05. Error bars show the standard deviation. See also Figure S4.

P1 and P2 fractions, with their high CFE, were cultivated in maturation medium and compared by *en face* observation and gene expression analysis (Figure 4A). After four weeks, homogeneous epithelial cell sheets had formed (Figure 4B), with those derived from P1 revealing the presence of MUC5AC (a goblet cell marker) and K13 (a conjunctival epithelial cell marker) (Figure 4C). Immunostaining of the epithelial sheet derived from P2, on the other hand, revealed the presence of K12 (a corneal epithelial cell marker) (Figure 4C). Gene expression analysis of P1 and P2 before maturation (i.e. FACS group) indicated that the expression levels of conjunctival differentiation markers such as *K13*/*K4*, mucin markers such as *MUC5AC*/*MUC4*, and corneal differentiation markers such as *K12*/*K3* were not elevated (Figure 4D). After maturation (i.e. Sheet group), however, conjunctival differentiation markers (*K13* and *K4* as conjunctival epithelial cell markers), *MUC5AC* (a goblet cell marker), *K7* (a unicellular gland marker), and *MUC4* (a membrane mucin marker) were highly expressed in the sheet derived from P1. Corneal differentiation markers (*K12* and *K3* as corneal epithelial cell markers) were highly expressed in the sheets derived from P2. Ocular surface differentiation markers (*PAX6* and *p63* as ocular surface epithelial cell markers) were expressed in sheets from both P1 and P2 (Figure 4E). Although this result was obtained from using the YZWJs524 hiPSC line, we have also confirmed, using line 201B7, that the expression of *K13* (a conjunctival epithelial cell marker) and *MUC5AC* (a goblet cell marker) was detected in the sheets derived from P1 (Figures S4D–S4F).

### Immunostaining of epithelial sheets fabricated from hiPSC-derived BST2+ cells

**Figure 5:**
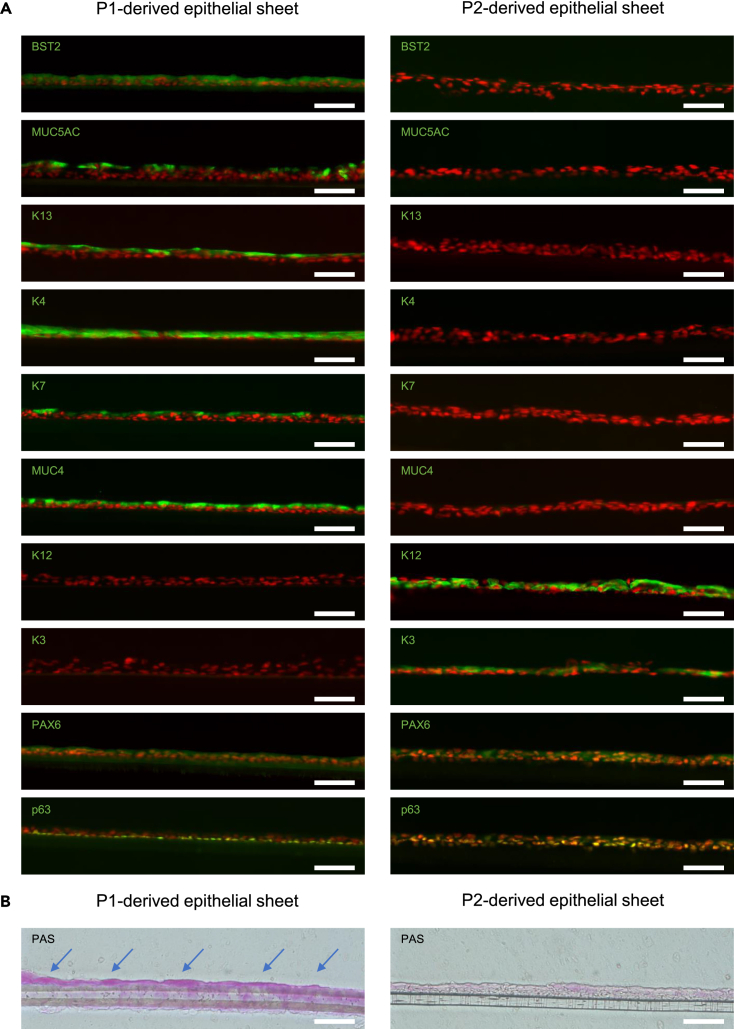
Immunostaining and PAS staining of epithelial sheets fabricated from hiPSC/SEAM-derived BST2+ cells (A) Immunostaining for ocular surface epithelial cell markers (green) in the BST2+(P1)-derived epithelial sheets are shown in the left panels, with BST2-(P2)-derived epithelial sheets on the right. n = three independent experiments. Nuclei, red. Scale bars; 50 μm. (B) PAS staining of the P1-derived and P2-derived epithelial sheet. PAS-positive cells are indicated with arrows in the P1-derived epithelial sheet. n = three independent experiments. Scale bars; 50 μm.

The immunostaining characteristics of P1-derived and P2-derived epithelial sheets were compared (Figure 5A). This showed that the goblet cell marker MUC5AC, the conjunctival epithelial cell markers K13 and K4, the membrane-bound mucin MUC4, and the unicellular gland marker K7, all of which are characteristic of the conjunctival epithelium, were present in the P1-derived epithelial sheets. In contrast, positive staining for the corneal epithelial cell markers K12 and K3 was seen in P2-derived epithelial sheets. Both P1- and P2-derived sheets were immunopositive for the ocular surface epithelial cell markers PAX6 and p63 (Figure 5A). PAS-positive cells were also identified in the P1-derived epithelial sheets, indicating the presence of mucin, with no PAS-positive cells observed in the P2-derived epithelial sheets (Figure 5B).

## Discussion

Numerous markers for corneal epithelial cells exist; however, conjunctival epithelial cell markers have seldom been reported and no cell surface marker has been discovered that can distinguish conjunctival epithelial stem/progenitor cells from corneal epithelial stem/progenitor cells, probably because the respective cell types are similar in nature. Now, however, we report six novel putative conjunctival epithelial cell markers—BST2, SLC2A3, AGR2, TMEM54, OLR1, and TRIM29—for example, one of which, AGR2, is known to be associated with intestinal and pulmonary goblet cells^17^^,^^18^ where it helps regulate goblet cell differentiation and protein glycosylation.^19^ AGR2 has not been reported previously in the conjunctival epithelium, a tissue that also contains mucin-producing goblet cells. Although we did not perform further experiments with these conjunctival epithelial cell markers with the exception of BST2, we believe that all these markers will help advance research on the human conjunctiva.

Further analysis ascertained that cultured conjunctival epithelial sheets containing goblet cells could be fabricated from conjunctival epithelial stem/progenitor cells that had been obtained from hiPSC-derived SEAM eye-like organoids and sorted by FACS using CD104, CD200, and one of the new conjunctival epithelial cell markers, BST2. BST2, also referred to as HM1.24, tetherin, or CD317, is a lipid raft-associated type II transmembrane glycoprotein that localizes on the cell surface.^20^ It is expressed to varying degrees in all organs examined so far and has been detected in many specialized cell types, including pneumocytes, hepatocytes, the ducts of major salivary glands, kidney, pancreas, various epithelia, Paneth cells, Leydig cells, monocytes, bone marrow stromal cells, plasma cells, and the vascular endothelium.^21^ BST2 is reported to be highly expressed in various tumors and is involved in the proliferative potential of cancers such as esophageal, gastric, colorectal, and breast cancer.^22^^,^^23^ Specifically, BST2 expression was upregulated in these tumor tissues compared to adjacent non-tumorous tissues. Here, we performed CFA and confirmed the higher proliferative potential of hiPSC/SEAM-derived BST2+ cells compared to BST2− cells, implying that BST2+ cells are present in the stem/progenitor cell population of the conjunctival epithelium. BST2 was strongly positive on basal regions of the fornix conjunctiva; this agrees with reports of conjunctival epithelial stem/progenitor cells that were abundant in the fornix conjunctiva.^7^^,^^8^ We also discovered that BST2 is more abundant on the bulbar side of the fornix conjunctiva than on the palpebral side of the fornix conjunctiva. We therefore hypothesize that conjunctival epithelial stem/progenitor cells are also more abundant in the bulbar side of the fornix conjunctiva.

BST2 is also an innate immune protein that responds to enveloped viruses in mammals^24^ and plays an important role in the defense of the host cell by preventing the release and spread of various enveloped viruses from the infected cells. We speculated that the difference in BST2 distribution between the corneal epithelium and conjunctival epithelium might be because of the differences in the immune responses of the respective epithelia. For example, the conjunctiva contains organized lymphoid tissue comprising adjacent lymphocytes, subepithelial lymphoid follicles, intraepithelial lymphatics, and blood vessels. This conjunctiva-associated lymphoid tissue, which does not exist in the cornea, forms an immunological interface between the ocular surface and external environment,^25^ and protects against infection by enveloped viruses such as adenovirus.^26^ While most cases of conjunctivitis caused by an adenovirus are self-limiting, corneal inflammation caused by adenoviruses can continue or recur for a long term after infection, leading to reduced vision.^27^ Although the immune response of the ocular surface is not completely understood, differences in the respective responses of the cornea and conjunctiva to viral infection could be potentially related to the different expression of BST2 in the epithelia of the two tissues.

Previously, we succeeded in isolating conjunctival epithelial cells, including stem/progenitor cells, from hiPSC-derived SEAMs by conducting FACS with specific cell surface markers, SSEA4, CD104, and CD200.^12^ However, no available cell surface marker could accurately separate conjunctival epithelial stem/progenitor cells from corneal epithelial stem/progenitor cells. In the previous report, we used SSEA4, which was weakly expressed in the native conjunctival epithelium and strongly expressed in the native corneal epithelium, as a negative marker. This approach^12^ meant that some corneal epithelial stem/progenitor cells would invariably be mixed in with the conjunctival epithelial stem/progenitor cells. The new approach using BST2 is able to accurately separate conjunctival epithelial stem/progenitor cells from corneal epithelial stem/progenitor cells to enable the generation of a hiPSC/SEAM-derived conjunctival epithelial sheet with high purity. This represents an important advance for our fundamental understanding of conjunctival epithelial cell biology and has applications for regenerative medicine-based treatments of ocular surface diseases, as well as drug discovery research targeting the conjunctival epithelium.

### Limitations of the study

The present study demonstrates that BST2-positive cells give rise to both conjunctival epithelial cells and goblet cells. Conjunctival epithelial stem/progenitor cells are generally believed to possess the capability of multipotent differentiation into both cell types;^7^ however, the results we provide here do not provide direct evidence to definitively confirm the multipotency of BST2-positive cells.

## STAR★Methods

### Key resources table

**Table:** 

REAGENT or RESOURCE	SOURCE	IDENTIFIER
**Antibodies**		

Mouse monoclonal anti-p63	Santa Cruz Biotechnology	Cat# sc-8431; RRID: AB_628091; clone 4A4
Rabbit polyclonal anti-PAX6	Covance Research Products Inc	Cat# PRB-278P; RRID: AB_291612
Goat polyclonal anti-K12	Santa Cruz Biotechnology	Cat# sc-17098; RRID: AB_639266
Mouse monoclonal anti-MUC5AC	Santa Cruz Biotechnology	Cat# sc-33667; RRID: AB_627973; clone CLH2
Mouse monoclonal anti-K13	Abcam	Cat# ab16112; RRID: AB_302267; clone AE8
Mouse monoclonal anti-MUC4	Abcam	Cat# ab52263; RRID: AB_881163; clone 8G-7
Mouse monoclonal anti-K4	Abcam	Cat# ab9004; RRID: AB_306932; clone 6B10
Mouse monoclonal anti-K7	Abcam	Cat# ab9021; RRID: AB_306947; clone RCK105
Mouse monoclonal anti-K3	PROGEN Biotechnik	Cat# 61807; RRID: AB_2133736; clone AE5
Rabbit polyclonal anti-BST2	Atlas antibodies	Cat# HPA017060; RRID: AB_1845467
Rabbit polyclonal anti-SLC2A3	Atlas antibodies	Cat# HPA006539; RRID: AB_1078984
Rabbit polyclonal anti-AGR2	Abcam	Cat# ab43043; RRID: AB_2225120
Rabbit polyclonal anti-TMEM54	Atlas antibodies	Cat# HPA061992; RRID: AB_2684652
Rabbit polyclonal anti-OLR1	Proteintech	Cat# 11837-1-AP; RRID: AB_2299037
Mouse monoclonal anti-TRIM29	Santa Cruz Biotechnology	Cat# sc-376125; RRID: AB_10989362
Mouse monoclonal anti-CD317 PE conjugate	BioLegend	Cat# 348406; RRID: AB_10564402
Mouse monoclonal anti-SSEA-4 PE conjugate	BioLegend	Cat# 330406; RRID: AB_1089206; clone MC-813-70
Mouse monoclonal anti-CD104 Alexa Fluor 647 conjugate	BD Biosciences	Cat# 624024; clone 450-9D
Mouse monoclonal anti-CD200 PE-Cy7 conjugate	BD Biosciences	Cat# 624052; clone MRC OX-104
Mouse monoclonal anti-IgG1 kappa isotype Ctrl PE conjugate	BioLegend	Cat# 400112; RRID: AB_2847829
Mouse monoclonal anti-IgG3 kappa isotype Ctrl PE conjugate	BioLegend	Cat# 401320; RRID: AB_10683168
Mouse monoclonal anti-IgG1 kappa isotype Ctrl Alexa Fluor 647 conjugate	BD Biosciences	Cat# 565571; RRID: AB_2687590
Mouse monoclonal anti-IgG1 kappa isotype Ctrl PE-Cy7 conjugate	BD Biosciences	Cat# 557872; RRID: AB_396914
Donkey anti-mouse IgG Alexa Fluor 488 conjugate	Life Technologies	Cat# A-21202; RRID: AB_141607
Donkey anti-rabbit IgG Alexa Fluor 594 conjugate	Life Technologies	Cat# A-21207; RRID: AB_141637
Donkey anti-goat IgG Alexa Fluor 568 conjugate	Life Technologies	Cat# A-11057; RRID: AB_142581

**Biological samples**		

Research-grade human corneoscleral tissue	CorneaGen	https://corneagen.com/
Human conjunctival tissue	Osaka University Hospital	N/A
Cynomolgus monkey	Ina research	https://www.ina-research.co.jp/

**Chemicals, peptides, and recombinant proteins**		

iMatrix-511 (LN511E8)	Nippi	Cat# 892012
Fetal bovine serum (FBS)	Life Technologies	Cat# 12483-020
Mitomycin C	Kyowa Hakko Kirin	N/A
Dispase II, powder	Thermo Fisher Scientific	Cat# 17105-041
TrypLETM Express Enzyme (1X), phenol red	Thermo Fisher Scientific	Cat# 12605010
KnockOutTM Serum Replacement (KSR)	Life Technologies	Cat# 10828-028
Sodium pyruvate	Life Technologies	Cat# 11360-070
Non-essential amino acids	Life Technologies	Cat# 11140-050
L-glutamine	Thermo Fisher Scientific	Cat# 25030081
Penicillin-streptomycin solution	Life Technologies	Cat# 15140-122
2-Mercaptoethanol	Life Technologies	Cat# 21985-023
Y-27632	Wako	Cat# 034-24024
Recombinant human EGF	R&D Systems	Cat# 236-EG
Recombinant human KGF	Wako	Cat# 112-00813
B27 supplement	Life Technologies	Cat# 17504-044
Sepasol-RNA I Super G	Nacalai tesque	Cat# 09379-55
4% paraformaldehyde phosphate buffer solution	Wako	Cat# 163-20145
Optimal Cutting Temperature Compound	Sakura Fineteck	Cat# 45833
Normal donkey serum	Jackson ImmunoResearch	Cat# 017-000-121
Triton-X 100	Sigma-Aldrich	Cat# T8787
Hoechst 33342	Wako	Cat# 346-07951
StemPro Accutase Cell Dissociation Reagent	Life Technologies	Cat# A11105-01
10% formaldehyde neutral buffer solution	Wako	Cat# 062-01661
Rhodamine B	Wako	Cat# 180-00132
DMEM	Life Technologies	Cat# 11995-065
GMEM	Life Technologies	Cat# 11710-035
CnT-PR (without EGF and FGF2)	CELLnTEC Advanced Cell Systems	N/A
DMEM/F12	Life Technologies	Cat# 11320-033
Stem FIt	Ajinomoto	N/A
Phosphate-Buffered Saline (PBS)	Wako	Cat# 164-23551
Tris-Buffered Saline (TBS)	Takara Bio	Cat# T9141
Bovine serum albumin (BSA)	Invitrogen	Cat# AM2618

**Critical commercial assays**		

PAS staining kit	MERCK KGaA	Cat# 101646
PrimeScript™ RT Master Mix (Perfect Real Time)	TAKARA Bio	Cat# RR036A

**Deposited data**		

ScRNA-seq datasets (Conjunctival epithelial cells; CjEC)	NCBI SRA repository	Accession numbers: SRR23295651
ScRNA-seq datasets (Corneal epithelial cells; CEC)	NCBI SRA repository	Accession numbers: SRR23295650
ScRNA-seq datasets (Ocular surface epithelial cells; OEC)	NCBI SRA repository	Accession numbers: SRR23295649

**Experimental models: Cell lines**		

Human: iPS cell line YZWJs524	Center for iPS Cell Research and Application	N/A
Human: iPS cell line 201B7	RIKEN Bio Resource Center	Cat# HPS0063; RRID: CVCL_A324
Mouse: NIH 3T3 cell line	N/A	N/A

**Oligonucleotides**		

SYBR probes were listed in Table S1	N/A	N/A

**Software and algorithms**		

AxioVs40 version 4.8.2.0	Carl Zeiss	N/A
7500 Software version 2.0.6	Thermo Fisher Scientific	RRID: SCR_014596; https://www.thermofisher.com/us/en/home/technicalresources/software-downloads/appliedbiosystems-7500-real-time-pcrsystem.htm
Cell Sorter Software version 2.1.5	SONY	N/A
JMP pro version 17.1.0	SAS	RRID: SCR_014242; https://www.jmp.com/en_us/software/predictive-analytics-software.html
Chromium Next GEM Single cell 3′ kit	10x Genomics	Cat# 1000092
Cell Ranger Software version 3.0.2	10x Genomics	N/A
R package Seurat version 3.0.0	10x Genomics	https://satijalab.org/seurat/pbmc3k_tutorial.html
Velocyte version 0.17	10x Genomics	N/A

### Resource availability

#### Lead contact

Further information and requests for resources and reagents should be directed to and will be fulfilled by the Lead Contact, Ryuhei Hayashi (ryuhei.hayashi@ophthal.med.osaka-u.ac.jp)

#### Materials availability

This study did not generate new unique reagents.

### Experimental model and study participant details

#### hiPSC lines

The hiPSC line YZWJs524 was obtained from Kyoto University’s Center for iPS Cell Research and Application Foundation.^28^^,^^29^ 201B7 hiPSCs were obtained from RIKEN Bio Resource Center (Tsukuba, Japan).^30^ Cells were cultured in serum-free medium (StemFit; Ajinomoto, Tokyo, Japan) on laminin-511 E8 fragments (LN511E8; Nippi, Tokyo, Japan)-coated dishes (0.5 μg/cm^2^) at 37°C.^31^ All experiments using recombinant DNA were approved by the Recombinant DNA Committee of Osaka University.

#### Human corneal and conjunctival epithelia

Research-grade donor human corneoscleral tissue was obtained from CorneaGen (Seattle, WA, USA) from which biopsies of corneal tissue were excised using surgical micro-scissors. Conjunctival tissue was obtained from patients undergoing conjunctiva chalasis surgery at Osaka University Hospital, with informed consent. The excess bulbar conjunctiva was dissected away and used for our research projects. Ethics committee approval for this work was obtained from Osaka University Hospital (permission #21191).

The corneal and conjunctival tissue specimens were separately immersed in DMEM (Life Technologies, Carlsbad, CA, USA) containing Dispase (Thermo Fisher Scientific, Waltham, MA, USA) for 60 min at 37°C, after which the epithelial layers of each tissue were scraped with forceps and dissociated for 20 min at 37°C using TrypLE TM Express Enzyme (Thermo Fisher Scientific). Human corneal and conjunctival epithelial cells were thus obtained.

#### Conjunctival epithelium from cynomolgus monkey

Two adult cynomolgus monkeys (a 4-year-old female and a 5-year-old female) were obtained from Ina Research (Ina, Japan) and after euthanasia, conjunctival tissue was surgically obtained and divided into bulbar, fornix and palpebral conjunctiva. All animal care and experimental protocols conformed to the Guidelines for Animal Experiments of Osaka University and were approved by Animal Experimental Committee of Osaka University.

### Method details

#### Differentiation of hiPSCs using the SEAM method

The fabrication procedure to generate SEAMs from hiPSCs is shown in Figure 1A and was performed as previously described.^10^^,^^12^ Briefly, hiPSCs were seeded onto LN511E8-coated plates and cultured in StemFit medium for 10 days. The medium was then switched to differentiation medium (DM; GMEM (Life Technologies) containing 10% Knockout Serum Replacement (KSR; Life Technologies), 1 mM sodium pyruvate (Life Technologies), 0.1 mM non-essential amino acids (Life Technologies), 2 mM L-glutamine (Thermo Fisher Scientific), 1% penicillin-streptomycin solution (Life Technologies), and 55 μM 2-mercaptoethanol (Life Technologies). After four weeks of culture in DM, the medium was switched to ocular surface differentiation medium (ODM; diluted 1:1 in DM and Cnt-PR without EGF and FGF2 (CELLnTEC Advanced Cell Systems, Bern, Switzer land) supplemented with 10 μM Y-27632 (Wako, Osaka, Japan) and 1% penicillin-streptomycin solution). As required, 10 ng/mL EGF (R&D Systems, Minneapolis, MN, USA) or 10 ng/mL KGF (Wako) was added to the medium. Manual pipetting was performed to remove non-epithelial cells after three weeks of culture in ODM. After four weeks of culture in ODM, the medium was switched to ocular surface epithelium maintenance medium (OEM; DMEM/F12 (Life Technologies) supplemented with 10 μM Y-27632, 2% B-27 supplement (Life Technologies) and 1% penicillin-streptomycin solution). As required, 10 ng/mL EGF or 10 ng/mL KGF was added to the medium. After four weeks of culture in OEM (i.e. the total time in culture was 12 weeks), the cultured cells were subjected to flow cytometry and cell sorting. In all experiments, the plates were incubated at 37°C in a 5% CO_2_ incubator. Phase-contrast microscopy was performed using an Axio Observer D1 (Carl Zeiss, Oberkochen, Germany) and analyzed with AxioVs40 software (Carl Zeiss).

#### Fluorescent activated cell sorting (FACS)

SEAM organoids cultured for 12 weeks were analyzed using FACS based on previous reports.^10^^,^^12^ Cells were dissociated using Accutase (Life Technologies) for 30–60 min at 37°C and re-suspended in an ice-cold keratinocyte culture medium (KCM) supplemented with 5% fetal bovine serum (FBS; Life Technologies). The harvested cells were then stained with antibodies against PE-conjugated anti-SSEA4 (MC813-70, BioLegend, San Diego, CA, USA) or PE-conjugated anti-CD317 (BST2, Tetherin) (RS38E, BioLegend), Alexa Fluor 647 (AF647)-conjugated anti-CD104 (ITGB4) (450-9D, BD Biosciences, San Diego, CA, USA) and PE-Cy7 conjugated-anti-CD200 (OX-104, BD Biosciences) for 60 min on ice. After washing twice with phosphate-buffered saline (PBS) (Wako), the stained cells were sorted using an SH800 instrument (SONY Biotechnology, Tokyo, Japan). In all experiments, the harvested cells were stained with isotype antibodies corresponding to each antibody, as in the control experiments (BioLegend and BD Biosciences). Data were analyzed using the Cell Sorter software (SONY Biotechnology).

#### Culture of sorted hiPSCs

Cells isolated by cell sorting (P1-P2) were seeded on LN511E8-coated (0.5 μg/cm^2^) dishes at a density of 1.0 x 10^4^ cells/cm^2^ in cell culture inserts and cultured in maturation medium (DMEM/F12 supplemented with 10 mM Y-27632, 2% B27 supplement, 1% penicillin-streptomycin solution, and 20 ng/mL KGF) for 3–4 weeks according to previously described methods.^10^^,^^12^

#### Immunofluorescence staining

Human and cynomolgus monkey tissue and hiPSC/SEAM-derived cell sheets were embedded in Optimal Cutting Temperature Compound (Sakura Fineteck, Tokyo, Japan) and sectioned at 10 μm thickness. The sections were fixed in 2–4% paraformaldehyde (Wako), washed three times in Tris-buffered saline (TBS) (TaKaRa Bio, Shiga, Japan) for 5 min, and incubated for 1 h in TBS containing 5% normal donkey serum (Jackson immunoReseach, West Grove, PA, USA) and 0.3% Triton X-100 (Sigma-Aldrich, St. Louis, MO, USA) to block non-specific reactions. Next, cells/tissues were incubated with the primary antibodies listed in the key resources table at 4°C overnight and stained with Hoechst 33342 (Wako) and Alexa Fluor-conjugated secondary antibodies (Life Technologies) for 1 h at room temperature. Stained cells or tissues were observed using an Axio Imager D1 (Carl Zeiss).

#### PAS staining

Tissue slides of P1-derived epithelial sheets and P2-derived epithelial sheets were fixed with 10% formaldehyde neutral buffer solution (Wako). The sections were stained using a PAS staining kit (MERCK KGaA, Darmstadt, Germany) in accordance with the manufacturer’s instructions. The stained sections were observed using an Axio Imager A2 microscope (Carl Zeiss).

#### Colony-forming assay (CFA)

Cells isolated by cell sorting (P1-P4) were seeded in 12-well plates onto mitomycin C-treated NIH 3T3 feeder cells at a density of 500–2,000 cells/well in KCM supplemented with 5% FBS, 10 ng/mL KGF, and 10 mM Y-27632. After cultivation for 10–14 days, the colonies were fixed with 10% formaldehyde neutral buffer solution then stained with rhodamine B (Wako). The colony-forming efficiency (CFE) was assessed by counting the colonies under a dissecting microscope.

#### Gene expression analysis

Total RNA was extracted from cells using Sepazol (Nacalai Tesque, Kyoto, Japan) and cDNA was synthesized using Prime Script RT Master Mix (Takara Bio). Quantitative reverse transcription-polymerase chain reaction (qRT-PCR) was performed using an ABI Prism 7500 Fast Sequence Detection System (Thermo Fisher Scientific) in accordance with the manufacturer’s instructions. A list of SYBR probes is provided in Table S1. The thermocycling program comprised an initial cycle at 95°C for 20s, followed by 45 cycles at 95°C for 3s and 60°C for 30s.

#### Single-cell RNA-sequencing

Cells isolated from hiPSC-derived SEAMs were washed twice with PBS supplemented with 0.04% bovine serum albumin (BSA) (Invitrogen, Waltham, MA, USA) and re-suspended in the same buffer at the density of 1.0 × 10^6^ cells/ml. The cells were processed with a Chromium Next GEM Single Cell 3′ kit (10x Genomics, Pleasanton, CA, USA) according to the manufacturer’s instructions. In brief, cell suspensions were loaded onto a Chromium single-cell controller (10x Genomics) to generate single-cell gel bead-in-emulsions. Reverse transcription was then performed, generating cDNA tagged with a cell barcode and a unique molecular index (UMI), followed by barcoded cDNA amplification and library construction. Libraries were sequenced on an Illumina HiSeq sequencing platform using paired-end sequencing with 30 cycles for read 1 and 90 cycles for read 2.

#### Quality control and processing of single cell RNA sequencing data

Cell Ranger Software version 3.0.2 (10x Genomics) was used to process 3′ Chromium scRNA-seq data. The reads were aligned to the Cell Ranger human reference genome (refdata-cellranger-GRCh38-3.0.0, 10x Genomics) and raw gene expression matrices were generated using “cellranger count”. The following data analysis steps were performed using R package Seurat version 3.0.0 according to the Seurat tutorial: (i) Genes expressed in three or more cells were retained. Cells with fewer than 200 genes or more than 6,000 genes and those with more than 20% mitochondrial genes were excluded. (ii) The data were normalized using the “NormalizeData” function: the UMI count for each gene from each cell was divided by the total UMI counts for the cell, multiplied by 10,000 scale factor and log-transformed. (iii) Highly variable genes were identified using the “FindVariableGenes” function with default parameters. A linear transformation was applied as a standard pre-processing step using the “ScaleData” function. (iv) Principal component analysis (PCA) for dimensionality reduction was performed using the “RunPCA” function, after which the first five computed PCs were used to perform unsupervised cell clustering using the “FindNeighbors” and “FindClusters” functions for use in t-distributed stochastic neighbor embedding (t-SNE). Differentially expressed genes were identified for each cluster using the Wilcoxon rank sum test implemented in Seurat. The RNA velocity analyses were performed based on Cellranger bam files. The spliced and unspliced UMIs for each gene in each cell were calculated using Velocyte (10x Genomics).

### Quantification and statistical analysis

All data are presented as the mean ± the standard deviation; n represents the number of biological replicates. The Wilcoxon rank-sum test (two-sided) was performed for two-group comparisons. Statistical significance was set to p < 0.05. All statistical analyses were performed using JMP Pro software (v17.0).

## Data Availability

• Single cell RNA-sequencing datasets have been deposited in the NCBI SRA and are publicly available as of the date of publication. Accession numbers are listed in the key resources table. • This paper does not report original code. • Any additional information required to reanalyze the data reported in this paper is available from the lead contact upon request.
