# Supplementary material for: Identification of BST2 as a conjunctival epithelial stem/progenitor cell marker
Source: iScience. 2023 Jun 5;26(7):107016. doi: 10.1016/j.isci.2023.107016 (PMC10300367; doi:10.1016/j.isci.2023.107016)
Supplement: Document S1. Figures S1–S4 and Table S1 [file mmc1.pdf]

iScience, Volume *26*

Supplemental information

Identification of BST2 as a conjunctival epithelial stem/progenitor cell marker

Masahiro Kitao, Ryuhei Hayashi, Kimihito Nomi, Reiko Kobayashi, Tomohiko Katayama, Hiroshi Takayanagi, Akiko Oguchi, Yasuhiro Murakawa, and Kohji Nishida

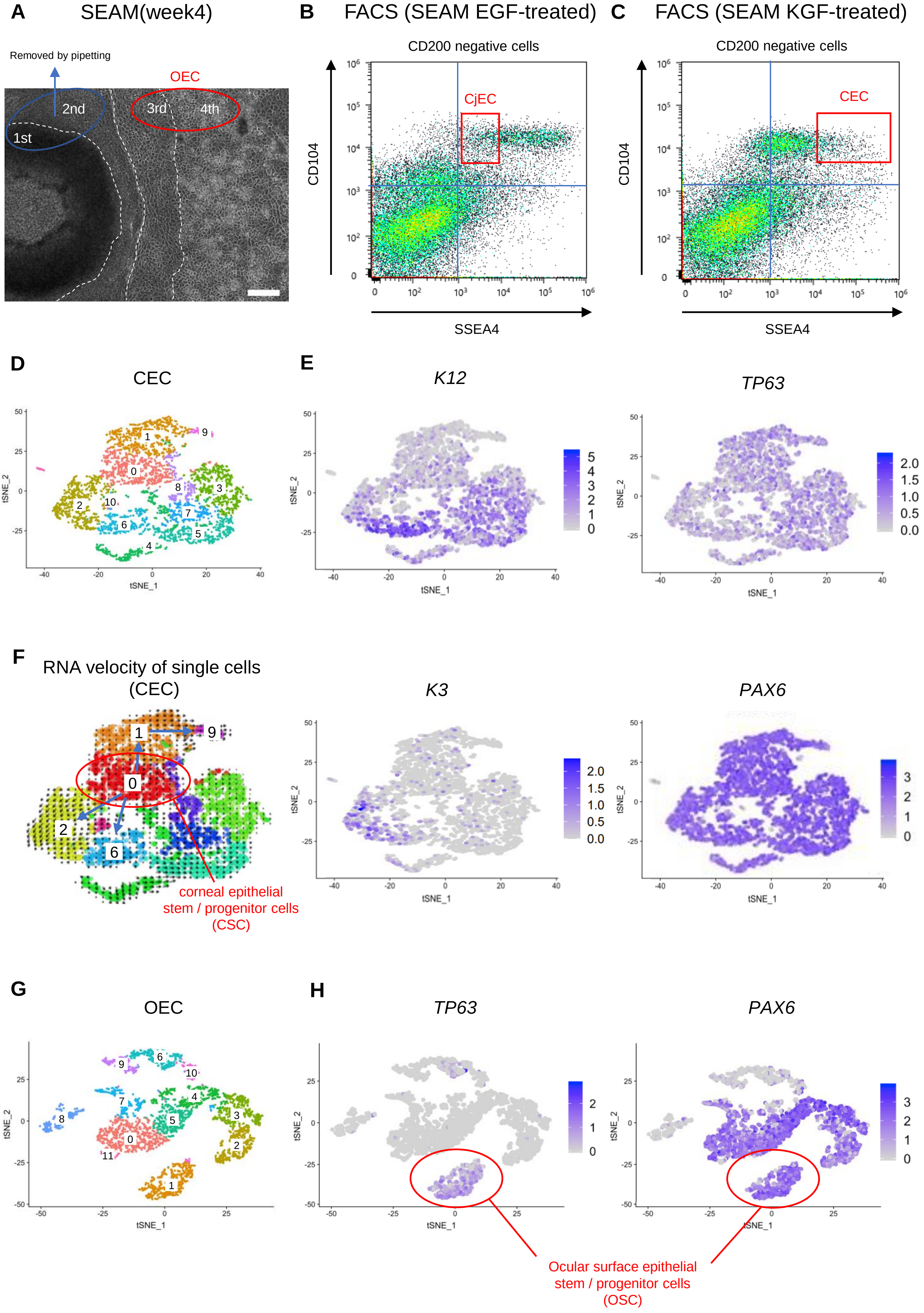

A
SEAM(week4)
Removed by pipetting
OEC
1st
2nd
3rd
4th
B
FACS (SEAM EGF-treated)
CD200 negative cells
CjEC
CD104
SSEA4
C
FACS (SEAM KGF-treated)
CD200 negative cells
CEC
CD104
SSEA4
D
CEC
tSNE_1
tSNE_2
E
K12
TP63
F
RNA velocity of single cells (CEC)
corneal epithelial stem / progenitor cells (CSC)
K3
PAX6
G
OEC
H
TP63
PAX6
Ocular surface epithelial stem / progenitor cells (OSC)

Figure S1. Single-cell RNA sequencing of corneal epithelial cells and ocular surface epithelial cells derived from hiPSCs via SEAMs, related to Figure 1.
A. The SEAM structure of hiPSCs at week 4, with four zones indicated, corresponding to ocular development. Scale bar: 50 μm. **B.** Flow cytometric analysis of SSEA4, CD200, and CD104 for the SEAM induced by EGF at week 12. **C.** Flow cytometric analysis of SSEA4, CD200, and CD104 for the SEAM induced by KGF at week 12. **D.** t-distributed stochastic neighbor embedding of corneal epithelial cells analyzed by single-cell RNA sequencing. **E.** Feature plots of K12, K3, TP63 and PAX6 of corneal epithelial cells. **F.** RNA velocity of single cells of corneal epithelial cells. Each arrow shows the direction of differentiation. **G.** t-distributed stochastic neighbor embedding of ocular surface epithelial cells analyzed by single-cell RNA sequencing. **H.** Feature plots of TP63 and PAX6 of ocular surface epithelial cells.

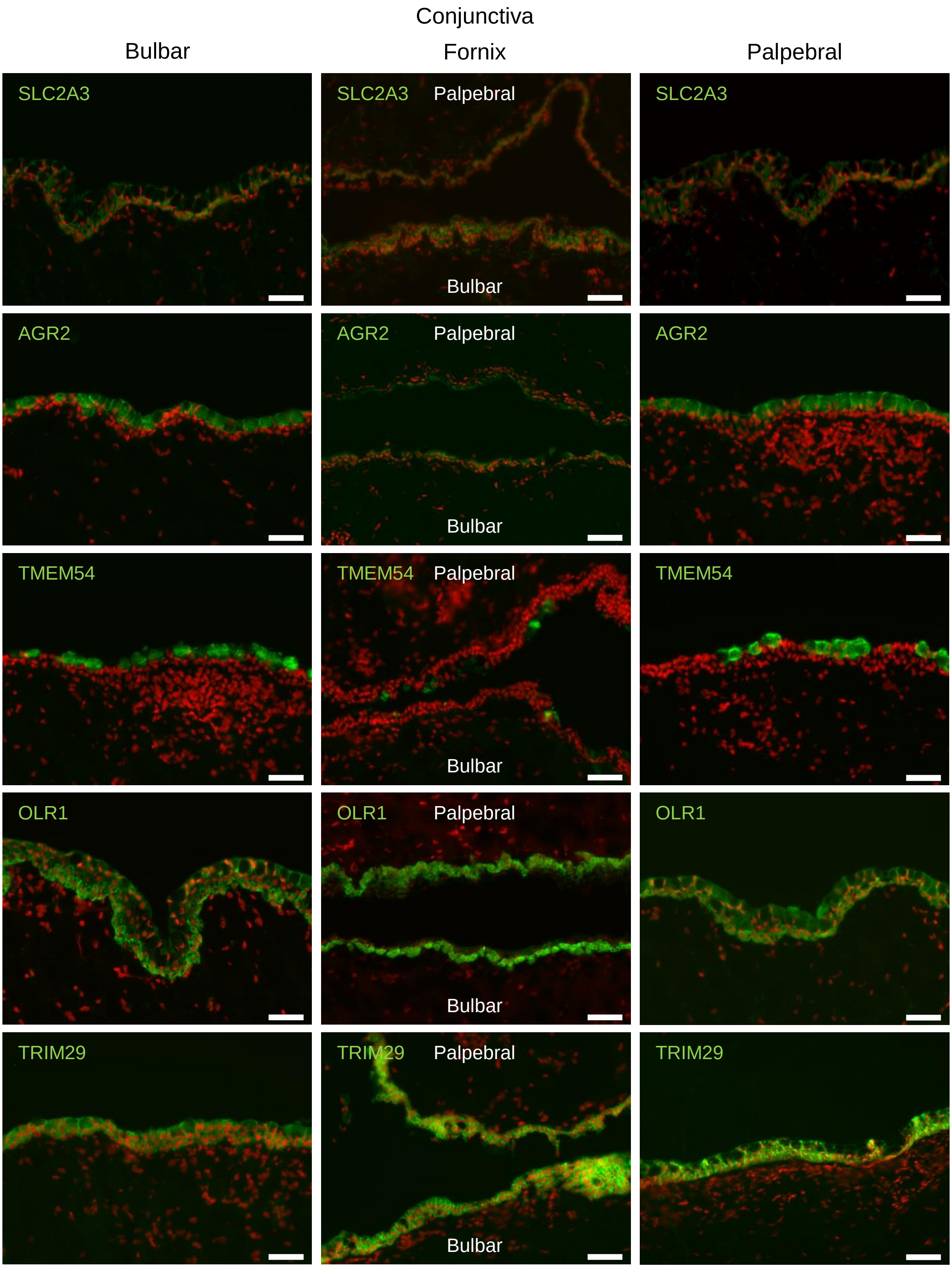

Figure S2. Novel conjunctival epithelial cell markers, related to Figure 2. Immunostaining of the bulbar, fornix, and palpebral conjunctiva of cynomolgus monkey with antibodies to SLC2A3, AGR2, TMEM54, OLR1, and TRIM29 (green). Nuclei, red. Scale bars; 50 μm.

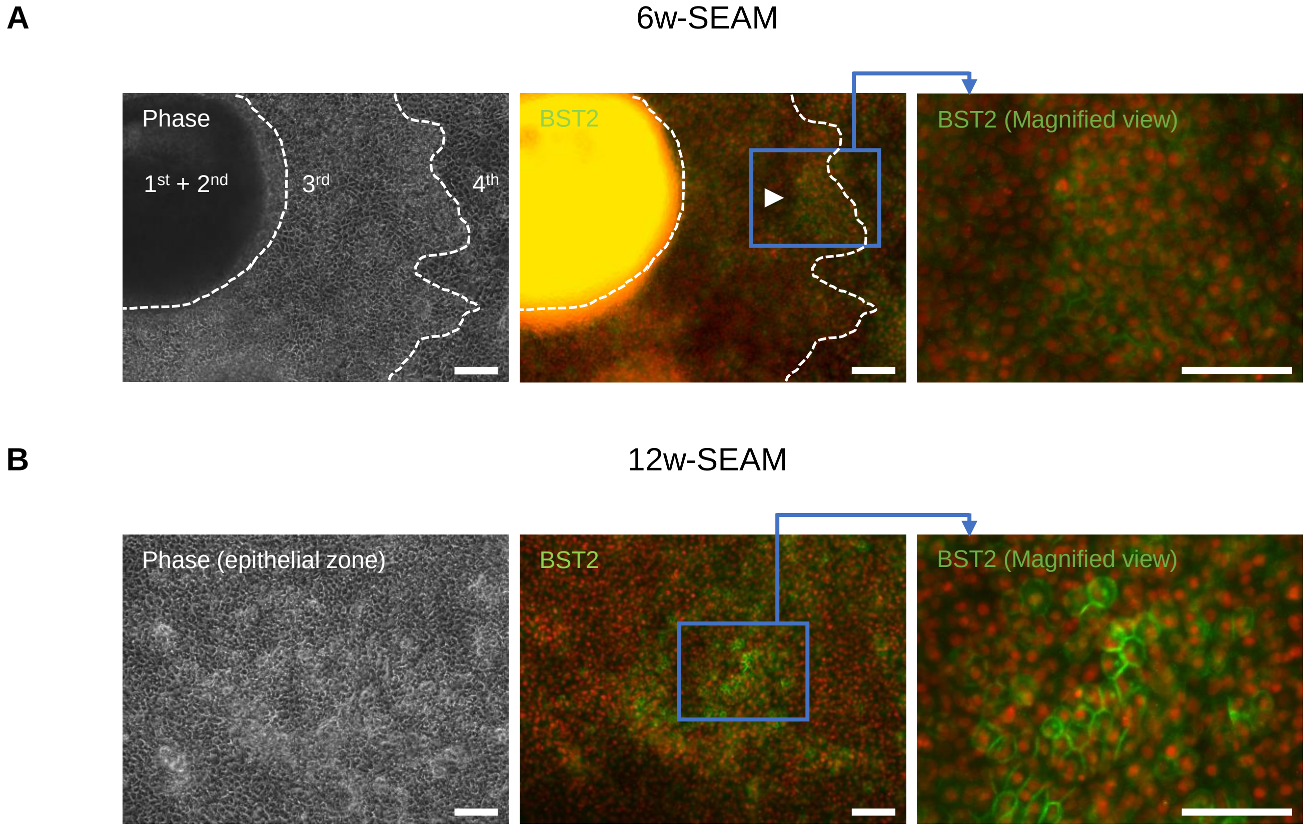

Figure S3. Immunostaining of the EGF-treated SEAMs at week 6 and week 12, related to Figure 3. **A.** Immunostaining for BST2 (green) in the EGF-treated SEAMs at week 6. Nuclei, red. Scale bars; 100 μm. **B.** Immunostaining for BST2 (green) in the EGF-treated SEAMs at week 12. Nuclei, red. Scale bars; 100 μm.

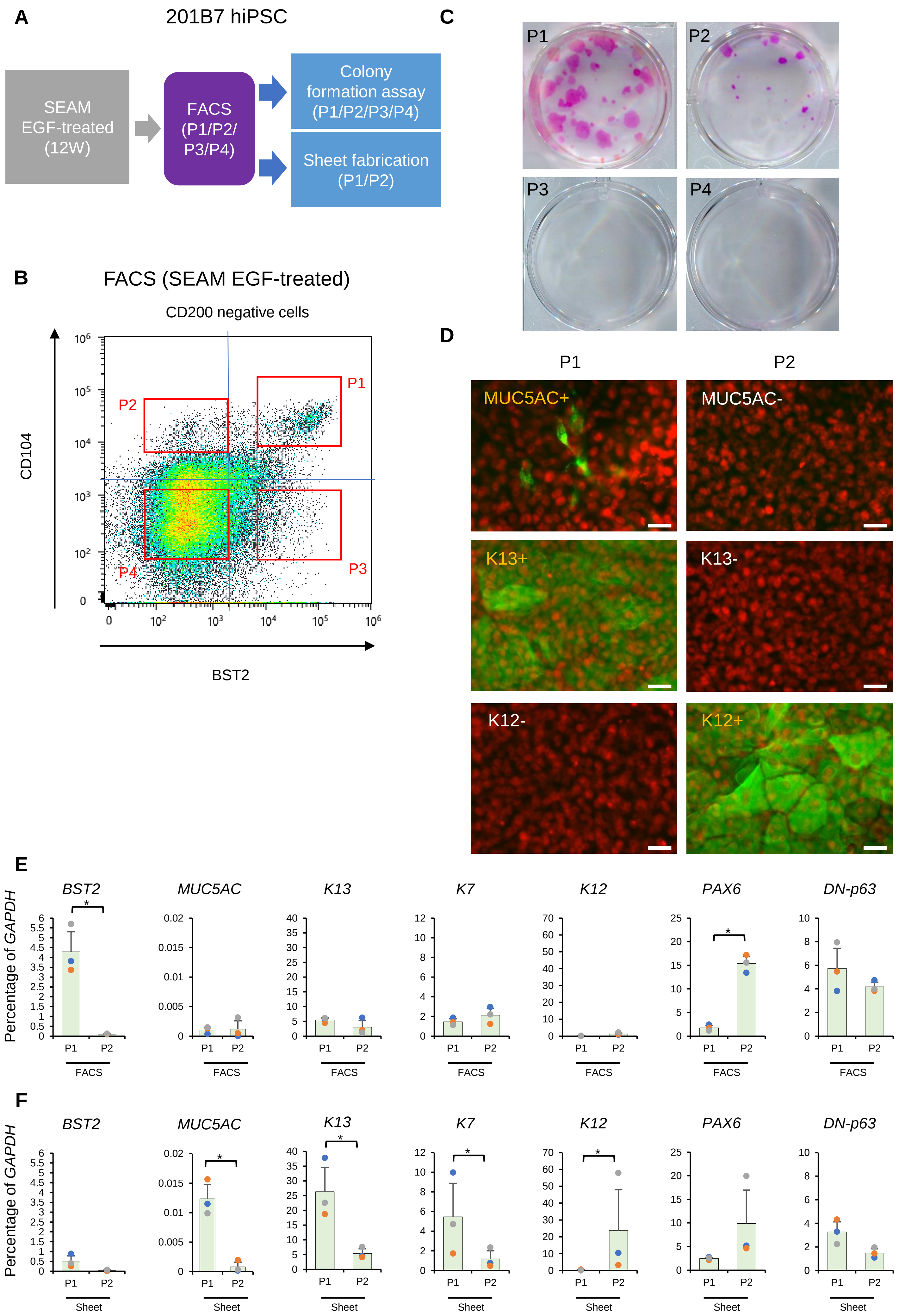

A
201B7 hiPSC
SEAM EGF-treated (12W)
FACS (P1/P2/ P3/P4)
Colony formation assay (P1/P2/P3/P4)
Sheet fabrication (P1/P2)
B
FACS (SEAM EGF-treated)
CD200 negative cells
CD104
BST2
P1
P2
P3
P4
C
P1
P2
P3
P4
D
P1
P2
MUC5AC+
MUC5AC-
K13+
K13-
K12-
K12+
E
Percentage of GAPDH
BST2
MUC5AC
K13
K7
K12
PAX6
DN-p63
FACS
F
Percentage of GAPDH
BST2
MUC5AC
K13
K7
K12
PAX6
DN-p63
Sheet

Figure S4. Proliferative potential and fabrication of epithelial sheets of hiPSC/SEAM-derived BST2+ cells from different iPSC line ‘201B7’, related to Figures 3-4.

A. Schematic of the differentiation and sorting method for the cells. **B.** Flow cytometric analysis of CD200, CD104, and BST2 for the EGF-treated SEAM derivative at week 12. CD200 (-) cells were extracted first, then the P1–P4 fractions were sorted based on CD104 and BST2 expression. P1–P4 fractions are defined as follows: (P1) CD200-, CD104+ and BST2+ cells; (P2) CD200-, CD104+ and BST2- cells; (P3) CD200-, CD104- and BST2+ cells; (P4) CD200-, CD104- and BST2- cells. **C.** Representative images of colony-forming assays of sorted cells from P1, P2, P3 and P4. n = three independent experiments. **D.** Immunostaining of MUC5AC, K13, and K12 (green) in the sheet derived from P1–P2. n = three independent experiments. Nuclei, red. Scale bars; 25 μm. **E.** Gene expression analysis of ocular surface epithelial markers for sorted cells from P1–P2 (FACS). n = 3. $*p < 0.05$. Error bars show the standard deviation. **F.** Gene expression analysis of ocular surface epithelial markers for the sheet derived from P1–P2 (Sheet). n = 3. $*p < 0.05$. Error bars show the standard deviation.

Gene name	Primer pair	Sequence (5'->3')	Template strand	Length	Tm	GC%
**BST2**	Forward	TTAAGCGTGAGAATCGCGGA	Plus	20	59.83	50
	Reverse	ACGGACCTTCCAAGATGTGC	Minus	20	60.32	55
**DN-p63**	Forward	TCAATTTAGTGAGCCACAGTACAC	Plus	24	59.01	41.67
	Reverse	TGGAAGGACACGTCGAAACT	Minus	20	59.25	50.00
**GAPDH**	Forward	GGAGCGAGATCCCTCCAAAAT	Plus	21	59.86	52.38
	Reverse	GGCTGTTGTCATACTTCTCATGG	Minus	23	59.38	47.83
**KRT4**	Forward	TTGCAGAGCTCAACAGGATGAT	Plus	22	60.03	45.45
	Reverse	TAAGGGCATTCTCACCTCGCT	Minus	21	61.24	52.38
**KRT7**	Forward	CTCAGGACCCTCAATGAGACG	Plus	21	59.86	57.14
	Reverse	CATCTCCTCATACTGCGCCTT	Minus	21	59.93	52.38
**KRT3**	Forward	CCAGGAGCGGGAACAGATCA	Plus	20	61.62	60
	Reverse	TGAGATGGAACTTGTGCCCTG	Minus	21	60.27	52.38
**KRT12**	Forward	CTGCTGAGGACTTCAGGATGA	Plus	21	60	52.38
	Reverse	CTCGTTCAGGCTCTCGATCT	Minus	20	59.83	55
**KRT13**	Forward	ATTGGTTTCCCTTCCTCAGCA	Plus	21	59.57	47.62
	Reverse	GGCGACCAGAGGCATTAGAG	Minus	20	60.25	60
**MUC4**	Forward	GGGAGAGGTATCGCCCTGAT	Plus	20	60.54	60
	Reverse	CACTCGAGACGGTAGTTGGG	Minus	20	59.83	60
**MUC5AC**	Forward	TCAGGAACAGCTTCGAGGAC	Plus	20	59.4	55
	Reverse	GTAGTAGGTTCCCGGCTTCAC	Minus	21	60.13	57.14
**PAX6**	Forward	TCCTTCACATCTGGCTCCATGTT	Plus	23	61.91	47.83
	Reverse	ATGCAGGAGTATGAGGAGGTCT	Minus	22	60.09	50

Table S1. A list of SYBR probes, related to STAR Methods.
